# Supplementary material for: Deciphering conformational selectivity in the A2A adenosine G protein-coupled receptor by free energy simulations
Source: PLoS Comput Biol. 2021 Nov 24;17(11):e1009152. doi: 10.1371/journal.pcbi.1009152 (PMC8654218; doi:10.1371/journal.pcbi.1009152)
Supplement: S1 Table — (PDF) [file pcbi.1009152.s001.pdf]

Table S1: Calculated and experimental relative binding free energies (kcal/mol) of the pairs of agonist/antagonist compounds of the $A_{2A}AR$ (data corresponding to Figure 4 in the main text)

**(Ago -> Ant)**	ΔG_{R*}	**SEM**	ΔG_{R}	**SEM**	$\Delta\Delta G_{calc}$	**SEM**	$\Delta\Delta G_{calc}$	**SEM**
**NECA - Cyp-Ade**	27.98	1.55	20.58	1.31	7.4	2.02	8	0.5
**ADO - Cyp-Ade**	13.84	0.56	10.23	0.81	3.61	0.97	3.6	0.5
**10b - 10a**	11.45	0.21	11.3	0.62	0.15	0.59	2.4	0.4
**10d - 10c**	12.02	0.34	11.54	0.39	0.48	0.52	0.8	0.4
**10g - 10f**	12.32	0.21	12.51	0.44	-0.19	0.46	1.6	0.4
**10j - 10i**	11.47	0.17	11.05	0.59	0.42	0.54	0.4	0.4
**10l - 10k**	11.35	0.47	10.39	0.58	0.96	0.74	2	0.4
**10n - 10m**	13.5	0.56	10.48	0.47	3.02	0.73	2.4	0.4
**LUF5833 - 5834**	10.16	0.44	12.13	0.4	-1.97	0.64	0	0.7
**LUF5833 - 5835**	12.69	0.19	10.09	0.66	2.6	0.6	2	0.6
