# Supplementary material for: Deciphering conformational selectivity in the A2A adenosine G protein-coupled receptor by free energy simulations
Source: PLoS Comput Biol. 2021 Nov 24;17(11):e1009152. doi: 10.1371/journal.pcbi.1009152 (PMC8654218; doi:10.1371/journal.pcbi.1009152)
Supplement: S2 Table — The effect of the mutation on the change in efficacy is calculated by adding these two values, according to the thermodynamic cycle shown in Fig 5 (ΔΔGEC50). (PDF) [file pcbi.1009152.s002.pdf]

Table S2: Calculated effects of point mutations on the constitutive activity (expressed as the shift in the distribution between R and R*, $\Delta\Delta G_{R^* \rightarrow R}$), and on the relative affinity of each ligand for the active receptor ($\Delta\Delta G_{bind, R^*}$). The effect of the mutation on the change in efficacy is calculated by adding these two values, according to the thermodynamic cycle shown in Fig. 5 ($\Delta\Delta G_{EC50}$).

				CGS21680					LUF5834				
			Basal activity			Internal Efficacy change					Internal Efficacy		
						Calc	Experimental				Calc	Experimental	
Mutant	ΔG_R	ΔG_{R^*}	$\Delta\Delta G_{R^* \rightarrow R}$	$\Delta G_{R^*\text{-}L}$	$\Delta\Delta G_{bind,R^*}$	$\Delta\Delta G_{EC50}$	$\Delta\Delta G_{EC50}$	*fold EC_{50}	$\Delta G_{R^*\text{-}L}$	$\Delta\Delta G_{bind,R^*}$	$\Delta\Delta G_{EC50}$	$\Delta\Delta G_{EC50}$	*fold EC_{50}
T88$^{3.36}$A	-2.64 ± 0.29	1.34 ± 0.16	3.98 ± 0.33	3.51± 0.16	2.17 ± 0.23	6.15 ± 0.33	4.43	1700	-2.87±0.21	-4.21 ± 0.26	-0.23 ± 0.36	-0.13	0.8
S277$^{7.42}$A	-7.37 ± 0.59	-7.34 ± 0.06	0.03 ± 0.59	-5.62± 0.38	1.72 ± 0.38	1.75 ± 0.70	2.80	110	-9.31±0.36	-1.97 ± 0.36	-1.94 ± 0.69	-0.55	0.4

* Data extracted from [1].

Reference

1. Lane JR, Herenbrink CK, Van Westen GJP, Spoorendonk JA, Hoffmann C, IJzerman AP. A novel nonribose agonist, LUF5834, engages residues that are distinct from those of adenosine-like ligands to activate the adenosine A 2a receptor. Mol Pharmacol. 2012;81(3):475–87.
