# Supplementary material for: Deciphering conformational selectivity in the A2A adenosine G protein-coupled receptor by free energy simulations
Source: PLoS Comput Biol. 2021 Nov 24;17(11):e1009152. doi: 10.1371/journal.pcbi.1009152 (PMC8654218; doi:10.1371/journal.pcbi.1009152)
Supplement: Attachment — Submitted filename: Response-letter_A2a_conformational-selectivity-r1.pdf [file pcbi.1009152.s003.pdf]

UPPSALA
UNIVERSITET

Hugo Gutiérrez de Terán, PhD
Associate Professor

Department of Cell and Molecular Biology
Uppsala University, BMC, Box 596
SE-751 24 Uppsala, Sweden

August 27th, 2021

Michael Gilson
Guest Editor
Plos Computational Biology

Dear Dr. Gilson,

Thank you for reconsidering a revised version of our manuscript entitled "**Deciphering conformational selectivity in the A_{2A} adenosine G protein-coupled receptor by Free Energy simulations"** that we submitted for consideration as a research article in *Plos Computational Biology*.

The following is a point-by-point response to the three independent referees, which we appreciate and have read in detail, and motivated the revised version of the manuscript amending the points of criticism.

Reviewer #1 expressed 2 major and 2 minor points.

1- It's not clear from the manuscript how the two distinct "active" structures mentioned in the methods are used in the calculation. I find this confusing, especially since it is mentioned elsewhere in the manuscript that only an active and inactive structure is needed. Was one or two "active" structures used? Moreover, 2YDV is not really fully active, but has been termed intermediate. How is the intermediate nature of this conformation taken into account? In addition, 2YDV has four thermostabilizing mutations in the receptor (L48A^2.46, A54L^2.52, T65A^2.63 and Q89A^3.37) that are absent in the receptor in 5G53 (I believe these are also absent in 4EIY). How are these mutations taken into account, as they might affect the results? Why not use the 3QAK structure (assuming an intermediate/active-like conformation is desired), which I believe is wild type?

The referee is correct, we only used one (partly) active structure, PDB code 2YDV, and reverted the thermostabilizing mutations as correctly anticipated by the referee. We have amended the description of the Methods section accordingly (p26, first paragraph of the section). This setup (2YDV with reverted thermostabilizing mutations) is the most adequate given that the chemotype of the co-crystallized ligand is simpler that in 3QAK, where the agonist induces an opening of the extracellular loops due to the bulky N6 substitution, which most ligands that we study do not share. In addition, it proved previously a very useful framework to reproduce, with high correlation, the effect of point mutations to agonist binding on this same structure (Keränen et al, Plos One, 2014; Keränen et al, Chem Commun, 2015).

2- When discussing molecular mechanisms of mutations/ligands, it would be extremely helpful to have clear renderings that visually communicate these ideas. Currently, there is only one figure in the manuscript that shows 3D structures (of

UPPSALA UNIVERSITET

Hugo Gutiérrez de Terán, PhD
Associate Professor

Department of Cell and Molecular Biology
Uppsala University, BMC, Box 596
SE-751 24 Uppsala, Sweden

binding modes, in this case). I fear that, for anyone who is not already familiar with this system, it will be difficult to envision how these mutations/chemical modifications work by just looking at figure 3 and the various bar plots.

We appreciate this comment, and have now included a new set of three 3D panels in Figure 5 (A), illustrating the FEP simulations of the two sidechains (Thr88Ala, Ser277Ala) in the presence of the ribose-containing agonist CGS21680. This image should help the reader to follow the structural consequences of those mutations, highlighting the loss of polar interactions within the ribose binding pocket.

Additional comments:

1- It would be helpful to be more explicit when describing the system preparation. (…) What was the protonation state of D2.50, and how might the protonation state of this residue affect the results?

We have now included the requested details in the Methods section, first paragraph. As the referee correctly guesses, the inactive (4EIY) structure contains a sodium ion and a charged form of D2.50. The active-like state, on the other side, cannot accommodate a sodium ion though the protonated state of D2.50 was maintained charged, as suggested in our previous work (Gutiérrez-de-Terán, Structure, 2013).

2- Is 10 ps sampling per λ window really sufficient?

The answer is yes, in this case, and the justification to be found on the convergence of the simulations judged by the s.e.m. over 10 replica simulations (Values now in Supplementary Tables S1 and S2), as we now explicitly state on the methods section (p 28). Please note that the sampling time for the sidechain perturbation here considered was 8-10 ns (explanation on p 27 of methods), and this was thoroughly examined by us in recent papers cited in this manuscript (Jespers, J Cheminf., 2019; Jespers, JCTC, 2019).

Reviewer #2

- The first part of the results –study of SAR of a series of ligands– is hard to follow for a non-specialist in A2AAR pharmacology (…) Figure 4 is not very informative. One cannot easily judge the relevance of the similarities and differences in the bars, as the labels in the X axis do not mean much and one needs to constantly refer to Figure 2 to understand the significance of the data.

We understand the concerns of the referee and appreciate the advice to facilitate the reading for a non-expert on the pharmacology of adenosine receptors. Consequently, we have divided the bar plot into three zones, and included insets with the 2D formula of each of the three chemical scaffolds involved in each case, highlighting the

UPPSALA
UNIVERSITET

Hugo Gutiérrez de Terán, PhD
Associate Professor

Department of Cell and Molecular Biology
Uppsala University, BMC, Box 596
SE-751 24 Uppsala, Sweden

substitutions that are varied along the FEP simulations. We believe that this way the dataset of 10 datapoints, corresponding to the FEP analysis of 18 ligands, is more clearly presented and easier to follow.

It seems that only Figure 4 and 5A contain new data (two bar plots) –plus one supplementary table. This makes the manuscript feel 'light' on analysis.

The two figures summarize the data that support the main conclusions of the article, and were prepared to allow a global vision of the problematic covered, i.e. the conformational selectivity of a GPCR, modulated either by ligands of different pharmacology (Fig 4), either by receptor mutations (Fig 5). The data behind these figures accounts for roughly 0.75 μs MD simulations, covering two protein systems, 18 chemical ligands (of different classes, see new Table S1 in the supplementary material) and 2 protein mutations characterized with 2 ligands each (Table S2, already present in the previous version). The FEP methodology and average simulation times used here have been validated a number of times in several research publications in journals like *Plos Comp Biol., Angew. Chem., Chem Comun*., as well as more specialized (several *J. Med. Chem*.), see references.

This work presents an interesting way to use thermodynamic cycles and molecular simulations to study structure-activity relationships. However, the manuscript in its present form seems too specialized and more suited to a molecular pharmacology journal.

The "interesting use of thermodynamic cycles" is precisely the key point of our article, and we are glad that the referee appreciates it. In contrast, the referee claims that this is not within the scope of this journal, an opinion that we do not share: Plos Comp. Biol. contains indeed a number of articles covering computational pharmacology of GPCRs, like our original work described in: Boukharta et al., Plos Comp Biol, 2014 (65 citations in G. Scholar).

Minor
- I don't see the point of Figure 1A. It only says that there are two states of the receptor.

Panel A in Figure 1 illustrates the two states of the receptor, which are mapped with the different thermodynamic cycles (panels B and C) and presents them in a consistent color code (orange, inactive; blue, active). It is precisely intended to help the reader that is not familiar with GPCRs, in line of the previous comment of this referee.

- Grammar and syntax should be revised once more.

We have done so in this version, thank you for the suggestion.

UPPSALA
UNIVERSITET

Hugo Gutiérrez de Terán, PhD
Associate Professor

Department of Cell and Molecular Biology
Uppsala University, BMC, Box 596
SE-751 24 Uppsala, Sweden

- The authors might consider finding more descriptive names for the ligands that do not require checking constantly Figure 2.

We think that the new insets in Figure 4, as discussed in major point 1 of this referee, should do the job.

- The thermodynamic cycles (Figures 1BC and 5A) would be much more clear if they displayed the corresponding ΔG in the branches instead of cartoons. It would also help if they included the final formula for ΔΔG, for instance.

Thanks for the suggestion. We have included the different delta_G on the panels in Fig 1AB (it was already present on Fig 5), and the corresponding final formulas to solve each cycle are now in the text.

Referee #3

A number of interesting things claimed to come out of this approach, as exemplified in Figures 4 and 5:
1. The effect of a mutation on basal activity (R->R* in Fig 5). I find that this point (1) is convincing - this is very useful data and could find widespread application.

We are glad to read this opinion about one of the main points of the article.

2. Modelling ligand efficacy as a combination of ligand affinity for the active state R* weighted by the accessibility to R* (…) However, only two examples are given (T88A and S277A), and so the authors have a high probability of getting the correct result by chance. It would be more convincing if there were 2 more examples, with one extra mutation that changes basal activity and 1 extra mutation that does not.

We again appreciate the positive impression of the referee. The reason why we only selected these two mutants for this original study, is because they are fully characterized with experimental efficacy shifts of reference ligands, as opposed to other mutational reports, i.e. Magnani, PNAS, 2011, 105:10744). However, we should add that the effect of the two mutations is examined under different conditions, that is: in the presence of two different types of agonist ligand, yielding a total of four data points around these two mutations. The random probability to find a total qualitative agreement (loss or gain in ligand efficacy) between the calculations and experiments for the 4 datapoints is indeed as low as 1/2^4 ~ 0.06.

3. Modelling the activation of a GPCR using FEP/a thermodynamic cycle. This is a bold claim, but not too much is made of this in the manuscript as the effect is indirect.

UPPSALA
UNIVERSITET

Hugo Gutiérrez de Terán, PhD
Associate Professor

Department of Cell and Molecular Biology
Uppsala University, BMC, Box 596
SE-751 24 Uppsala, Sweden

The impression of the referee might come from a miscommunication on our side regarding this point: We actually do not claim we can model the activation of a receptor with thermodynamic cycles. What we can do is to model the *regulation* of receptor activation induced by point mutation or chemical modifications on an agonist molecule. We have clarified this point on the manuscript, page 22 under the Discussion: “It is worth mentioning that this model is not intended to model the activation pathway per se, but instead the variations in the activity of the receptor induced by different types of modulators (i.e., external ligands, mutations.” And on p 24 “To the best of our knowledge, this is the first time that the *regulation* of the activation of a GPCR”

4. Predict the pharmacological profile of a series of full and partial agonists / antagonists. I do not find this point (Fig. 4) convincing as some of the bars in Figure 4 are very small - so I am not convinced that there is real discrimination between between say 10a and 10b. It maybe that the data needs to be described more carefully.

The referee is correct at pointing the pair 10a – 10b as one where our model does not offer a clear discrimination between their pharmacological profile. Indeed, the experimental efficacy shifts within this scaffold (10) are rather mild, and still our model correctly predicts the tendency in most of the cases. We have rewritten the description of this data: “Our modeling shows a tendency on prolinol-containing compounds to favor the binding to the active receptor, as compared to their pyrrolidine substituted compounds, with the exception of the pairs and 10g/f and, to some extent, 10b/a where no significant conformational selection is calculated”. We have also added a sentence describing the advantages and limitations of our approach in the Discussion: “Indeed, the qualitative correlation between the calculated delta-delta-G and the delta-efficacy is achieved in up to 70% of the pair comparisons performed, including those where the agonist/antagonist profile differences are more significant (i.e. NECA, ADO as full agonists, or LUF5835 and compound 10n as the most potent partial agonists within their chemotype).”

On balance I am very much in favour of publication and I hope the authors will be able to make significant improvements to the clarity of their article

We appreciate the criticisms of this referee as they offer the opportunity to enhance the quality of this manuscript in a revised version.

Minor Comments

The labelling in Fig. 2 is confusing as 10a is an antagonist and 10b is a partial agonist - but the panel shows the structure of a and b to be the same. There is probably a better way to do this: inclusion of a dotted line as for LUF may help.
Changed as suggested

UPPSALA UNIVERSITET

Hugo Gutiérrez de Terán, PhD
Associate Professor

Department of Cell and Molecular Biology
Uppsala University, BMC, Box 596
SE-751 24 Uppsala, Sweden

p20/16 T288(3.36)... antagonist -bound conformation,..... What follows is a new theme regarding S277(7.42) and so the comma should be a semicolon (;).
Changed as suggested

p20/16 ligand internal efficacy is an interesting concept, but it is new and so difficult to get your head around it. I think it would be clearer if the corresponding equation were put in parentheses after every time the phrase is used (as it is not used very often) to help with reader comprehension. We also have predicted internal efficacy (p21/17) and mutational ligand efficacy (Fig. 5). The various incarnations of internal efficacy are not helpful but rather add to confusion - are they the same thing or not?
Thanks for pointing this out. We have revised the text and unified expressions regarding internal efficacy.

p22/18 we have thin and thick dashed bars - but we have dense bars in Fig. 5. The descriptions need to the consistent. Dense is probably better as we don't know whether the bars are blue or white so think and thick are interchangeable.
Changed as suggested

p22/p18. The increases and decreases can be a little difficult to follow as there is a delta delta so the sign convention gets somewhat diluted. Where it says a predicted increase in affinity of LUF5834 (~ -0.2 kcal/mol)...
I suggest adding the numerical value to the text would make it easier to confirm that we are looking at the right bars.
Done as suggested (in addition to indication to the supplementary table where all the numerical values are explicitly shown)

Somewhere, the authors should note that R* is an oversimplification
But this was already stated! The referee probably missed that, the first time we introduce R* (p4, introduction) we follow with "This relatively simplistic model becomes more realistic when considering the influence on the receptor equilibrium of chemical modulators"

p26/22 There is a reference to intermediate states - the authors should elaborate how these would be identified in such a framework - would it require an X-ray structure of an intermediate state?
We have rephrased as "One could expect that the precise structures of those states could be structurally revealed in the near future", irrespective of the structural biology technique.

p28/24. wrong use of shortly. Delete 'shortly,' and start 'This protocol...'
Changed as suggested

UPPSALA UNIVERSITET

Hugo Gutiérrez de Terán, PhD
Associate Professor

Department of Cell and Molecular Biology
Uppsala University, BMC, Box 596
SE-751 24 Uppsala, Sweden

p30/p26 8-10 ns is very short and requires some justification. It basically assumes no conformational changes, not even minor ones?
We have added the following sentence: "The sampling times have been repeatedly shown to be sufficient for conformational sampling at equilibrium, assuming that only minor local conformational changes occur (as is the case of the models here presented)"

Sincerely yours,

Hugo Gutiérrez de Terán
Department of Cell and Molecular Biology
Biomedical Center, Uppsala University,
Box 590, S-75124 Uppsala, Sweden

Phone: +46 18 471 5056 Mobile: +46 70 634 75921
Fax: +46 18 536971 E-mail: hugo.gutierrez@icm.uu.se
