# Supplementary material for: Deciphering conformational selectivity in the A2A adenosine G protein-coupled receptor by free energy simulations
Source: PLoS Comput Biol. 2021 Nov 24;17(11):e1009152. doi: 10.1371/journal.pcbi.1009152 (PMC8654218; doi:10.1371/journal.pcbi.1009152)
Supplement: Attachment — Submitted filename: Response-letter_A2a_conformational-selectivity-r2.pdf [file pcbi.1009152.s005.pdf]

UPPSALA
UNIVERSITET

Hugo Gutiérrez de Terán, PhD
Associate Professor

Department of Cell and Molecular Biology
Uppsala University, BMC, Box 596
SE-751 24 Uppsala, Sweden

October 28th, 2021

Michael Gilson, Guest Editor
Nir Ben-Tal, Deputy Editor
Plos Computational Biology

Dear Dr. Gilson,

Thank you for your positive response about the revised version of our manuscript entitled "**Deciphering conformational selectivity in the A_{2A} adenosine G protein-coupled receptor by Free Energy simulations"** to be potentially published a research article in *Plos Computational Biology*.

Following your letter, we have uploaded necessary supplementary information to reproduce the data in Figures 4 and 5 in a Zenodo repository (https://zenodo.org/record/5602896#.YXlGHZ5ByF4). These includes the raw data used to build the tables provided on SI and Figures 4 and 5. But most important we provide in a clearly constructed directory tree all the input files necessary to reproduce our data, using the standard, opensource code of Q, QligFEP and QresFEP software, which are all on the github repository since publication in 2018 and 2019 respectively. We have indicated this in the method section of the manuscript. We should note to the editor that the request of referee 1 to deposit all trajectory files is out of the capacity limits of these repositories (we are talking about Tb units here) and re-generating these trajectories is trivial with all the data provided (we even provide one script to run all necessary simulations with exactly the same parameters; note that this would require more than 15K CPU-h)

It follows the point-by-point response to the minor concerns of the two independent referees.

Sincerely yours,

Hugo Gutiérrez de Terán
Department of Cell and Molecular Biology
Biomedical Center, Uppsala University,
Box 590, S-75124 Uppsala, Sweden

Phone: +46 18 471 5056   Mobile: +46 70 634 75921
Fax: +46 18 536971   E-mail: hugo.gutierrez@icm.uu.se

UPPSALA UNIVERSITET

Hugo Gutiérrez de Terán, PhD
Associate Professor

Department of Cell and Molecular Biology
Uppsala University, BMC, Box 596
SE-751 24 Uppsala, Sweden

Reviewer #1 The manuscript is improved, and the authors have addressed many of my concerns.

However, regarding my comment on the structures used in the study, I think the authors should clearly state (in the manuscript) that they used an agonist-bound, but not G protein–bound, structure (which the authors themselves refer to as "pseudo-active" in the introduction) to represent the active state. I think this is all the more important because Fig. 1 shows a G protein–bound active receptor. I think the reader will assume that the active structure shown in Fig. 1 was used for the simulations (the structures used for the rendering should also be specified in the caption). Since a G protein–bound structure is available for this receptor, it would be important to briefly explain why that structure was not used as the active state.

As already requested by this referee, the previously revised version of the manuscript stated clearly, on the methods section, which structure we used along the simulations as representative of the A_{2A}AR active state (p 26). Following the comment in this second revision, we are glad to further expand that explanation on the methods section, as well as to indicate on the discussion section (p22) that we used an agonist-bound (not G-protein coupled) structure, together with a justification of this choice, emphasizing that our reductionist model of the binding site is not sensible to the use presence of the G-protein in the simulation.
We hope that this time the referee is convinced about the validity of the starting structures used for the purpose of the models here developed.

Trajectories and analysis code should be made available in a public repository such as Zenodo

We have now included the requested files to reproduce our data in the Zenodo repository:

https://zenodo.org/record/5602896#.YXlGHZ5ByF4

And indicated this at the end of the methods section. The repository includes all the initial structures used for each simulation, together with the input files, to reproduce our data using the open-source versions of QligFEP or QresFEP (both available under https://github.com/qusers/qligfep), running over the open-source MD code Q (https://github.com/esguerra/q6, repositories now indicated on the methods section). The Zenodo repository also includes all the scripts to perform the simulations and the required analysis as to report the raw data used to build Figure4 and Figure 5. The

UPPSALA
UNIVERSITET

Hugo Gutiérrez de Terán, PhD
Associate Professor

Department of Cell and Molecular Biology
Uppsala University, BMC, Box 596
SE-751 24 Uppsala, Sweden

numerical data behind these figures (Supplementary material), is also here included as raw data as a reference.

Referee #3: I am pleased that the authors have been permitted to resubmit to PLOS computational biology as this manuscript lies better here than in PLOS one.

This is a vastly improved manuscript, which I think should be accepted. I just have a minor comment on the legend of Fig. 1, which can be clearer. e.g. what is meant by the neutral effect of an antagonist/ Would delta G really be zero? I know what is intended, but it probably needs wording differently

We have rephrased the legend and Figure 1 accordingly, since the term “neutral” should apply indeed to the antagonist, accounting for the precise pharmacological “neutral antagonist”, meaning no preference for either conformation of the receptor; as opposed to the agonist, which has pronounced conformational selection for the active conformation (now, represented by a thinner line in the *reverse* process represented in the thermodynamic cycle in the Figure).
