# Supplementary material for: Deciphering conformational selectivity in the A2A adenosine G protein-coupled receptor by free energy simulations
Source: PLoS Comput Biol. 2021 Nov 24;17(11):e1009152. doi: 10.1371/journal.pcbi.1009152 (PMC8654218; doi:10.1371/journal.pcbi.1009152)
Supplement: Attachment — Submitted filename: Review2.docx [file pcbi.1009152.s004.docx]

Review_2

I am pleased that the authors have been permitted to resubmit to PLOS computational biology as this manuscript lies better here than in PLOS one.

This is a vastly improved manuscript, which I think should be accepted. I just have a minor comment on the legend of Fig. 1, which can be clearer. e.g. what is meant by the neutral effect of an antagonist/ Would delta G really be zero? I know what is intended, but it probably needs wording differently
